# Acupuncture for dry eye disease after recovery from COVID-19: A protocol for systematic review and meta-analysis

**DOI:** 10.1097/MD.0000000000031234

**Published:** 2022-10-28

**Authors:** Xiaofang Lu, Jian Li, Shuhan Ye, Runyi Wang, Yao Xiao, Lu Liu, Wanning Lan, Yijun Chen, Zongli Liao

**Affiliations:** a Guangzhou University of Chinese Medicine, Guangzhou, China; b National Clinical Research Center for Respiratory Disease, the First Affiliated Hospital of Guangzhou Medical University, Guangzhou, China; c Institute of Respiratory Health, Guangzhou Medical University, Guangzhou, China; d School of Medicine and Health, Guangzhou Pearl-River Vocational College of Technology, Guangzhou, China; e Department of Traditional Chinese Medicine, Xiniujiao Community Hospital in Jingxi Street, Guangzhou, China.

**Keywords:** acupuncture, COVID-19, dry eye disease, meta-analysis, protocol, systematic review

## Abstract

Dry eye disease (DED) is a condition occurring worldwide. Studies have found that coronavirus disease 2019 (COVID-19) patients may have persistent dry eye symptoms for weeks and months after recovery, and the prevalence of dry eye is higher in COVID-19 patients than in people without COVID-19 infection. As one of the common ophthalmic diseases, the clinical application of acupuncture in the treatment of DED is not widely used nowadays.

**Methods:**

According to the retrieval strategies, randomized controlled trials (RCT) on the acupuncture for DED after recovery from COVID-19 were obtained from Embase, The Cochrane Library, Web of Science, Chinese National Knowledge Infrastructure database, Chinese Biomedical Database, Chinese Science and Technology Periodical database, The WanFang database. Studies were screened based on inclusion and exclusion criteria, and the Cochrane risk bias assessment tool was used to evaluate the quality of the studies. The meta-analysis was performed using Review Manager (RevMan 5.3) and STATA 14.2 software. Ultimately, the evidentiary grade for the results will be evaluated.

**Results:**

The study will provide a high-quality and convincing assessment of the efficacy and safety of acupuncture for DED after recovery from COVID-19 and will be published in peer-reviewed journals.

**Conclusion:**

Our findings will provide references for future clinical decision and guidance development.

## 1. Introduction

Dry eye disease (DED) is a condition occurring worldwide which is an ophthalmic disease with high incidence at present. It refers to the abnormal tear quality, quantity and kinetics caused by many factors, leading to the decline of tear film homeostasis and ocular surface inflammation. The main symptoms include ocular discomfort, tearing and burning sensation and visual dysfunction.^[1]^ At present, with the popularity of video software and the coronavirus disease 2019 (COVID-19) pandemic, people’s work and study have gradually shifted from offline to online, resulting in the increasing prevalence of dry eye and the trend of the younger age. According to studies, the prevalence of dry eye is affected by diagnostic criteria, population, constitution and other factors, and it is found that the global prevalence of dry eyes ranges from 5% to 50%, which has become a key factor affecting the quality of life of people around the world and has important research significance.^[2]^

CUHK Medicine study^[3]^ have found that COVID-19 patients may have persistent dry eye symptoms for weeks and months after recovery, and the prevalence of dry eye is higher in COVID-19 patients than in people without COVID-19 infection. The study shows a higher prevalence of DED among post-COVID-19 patients.

The treatment of dry eye is mainly artificial tears, but the effect of this method is limited, and preservatives will aggravate the inflammation of the eye surface, lack of long-term treatment effect. In contrast, acupuncture in the treatment of ocular xerosis has the advantages of less risk, no drug dependence and adverse reactions, and has been gradually accepted by patients.^[4]^ Acupuncture has a history in treating dry eye since ancient times. At present, the reliability of its efficacy has been confirmed by many clinical studies.^[5]^ Shen Yu and other^[6]^ used acupuncture, Shengzhu and other acupoints combined with acupuncture treatment, the total effective rate was 87.1%, with obvious clinical effect, and confirmed that acupuncture with ion introduction is an effective treatment of DED.

The aim of this study was to systematically and comprehensively search the published randomized controlled trials (RCT) of acupuncture in the treatment of dry eye in order to better understand the efficacy and safety of acupuncture in treating DED after recovery from COVID-19.

## 2. Methods and Analysis

### 2.1. Study registration

This systematic review protocol has been registered in the PROSPERO (No. CRD42022351657). We will follow recommendations outlined in The Cochrane Handbook of Systematic Review of Interventions and the preferred reporting items for systematic reviews and meta-analysis protocol statement guidelines. If amendments are needed, we will update our protocol to include any changes in the whole process of research.

### 2.2. Inclusion criteria for study selection

#### 2.2.1. Types of studies.

This study will only include RCTs of acupuncture alone or combined with other interventions in the treatment of DED after recovery from COVID-19. We will focus on the language of clinical studies in Chinese and English. Since the RCT with acupuncture as the main intervention has certain difficulty in the implementation of blind method, we will not emphasize whether blind method is adopted in the execution of clinical research. Non-RCT, reviews, case reports, experimental study, and animal study will be excluded.

#### 2.2.2. Participants.

Patients who recovered from COVID-19 were included and met the clinically recognized diagnostic criteria for dry eye (national or international diagnostic criteria for dry eye) or the symptoms of dry eye. Both children and adults were included. The diagnosis of DED includes Chinese or international diagnostic criteria.^[7–10]^

#### 2.2.3. Types of interventions.

Treatment group interventions comprised acupuncture, and comparator groups intervention: artificial tears (drug type, name is not limited) treatment.

#### 2.2.4. Types of outcomes.

In this meta-analysis, the main outcome is Ocular Surface Disease Index. We used the Schirmer I test, tear breakup time, corneal staining, and complications as secondary outcome indicators.

### 2.3. Search strategy

#### 2.3.1. Electronic searches.

We will retrieve the following 8 databases: Embase, The Cochrane Library, Web of Science, Chinese National Knowledge Infrastructure database, Chinese Biomedical Database, Chinese Science and Technology Periodical database, The WanFang database. The retrieval time is from December 2019 to August 2022. The method of subject words combined with free words for retrieval will be applied, and the retrieval formula will be formulated and adjusted according to the characteristics of each database. The key search terms were as follows: DED, COVID-19, acupuncture, RCTs, and controlled clinical trials. The final retrieval strategy for PubMed will be ruled in conjunction with medical and uncontrolled terms following several pre retrieval, and will be shown in Table 1. Similar strategies will be applied to other databases after adjustment. The language of the literature is limited to both Chinese and English. We will also retrieve clinical trial registration platforms such as Clinical Trials.gov trials registry, Chinese Clinical Trial Registry, etc to track ongoing or completed clinical studies.

**Table 1:** Search strategy used in PubMed database.

#1 Dry eye syndrome
OR Dry Eye Disease OR Dry Eye Diseases
OR Dry Eye OR Dry Eyes OR Evaporative Dry Eye Disease
OR Evaporative Dry Eye Syndrome OR Evaporative Dry Eye
OR Dry Eye, Evaporative OR Evaporative Dry Eyes
#2 COVID-19 OR COVID 19
OR SARS-CoV-2 Infection OR Infection,SARS-CoV-2
OR SARS CoV 2 Infection OR SARS-CoV-2 Infections
OR 2019 Novel Coronavirus Disease OR 2019 Novel Coronavirus Infection
OR 2019-nCoV Disease OR 2019 nCoV Disease
OR 2019-nCoV Diseases OR Disease,2019-nCoV
OR COVID-19 Virus Infection OR COVID-19 Pandemics
OR COVID19 Pandemic OR 2019-nCoV Infections
OR COVID-19 Virus Diseases OR SARS Coronavirus 2 Infection
OR Disease 2019, Coronavirus
#3 acupuncture OR eye needle OR eye acupuncture
#4 Randomized controlled trial OR clinical study OR Clinical Trial OR Controlled study OR Controlled Trial OR Random*Control* study OR random* Control* Trial
#5 #1 AND #2 AND #3 AND #4

#### 2.3.2. Other searches.

Taking account of possible omission, not only the published studies in journals but also gray literatures will be retrieved, mainly through conference papers and references. If possible, we will try to contact the researchers to obtain the required research data.

### 2.4. Data collection and analysis

#### 2.4.1. Selection of studies.

**Figure 1.**
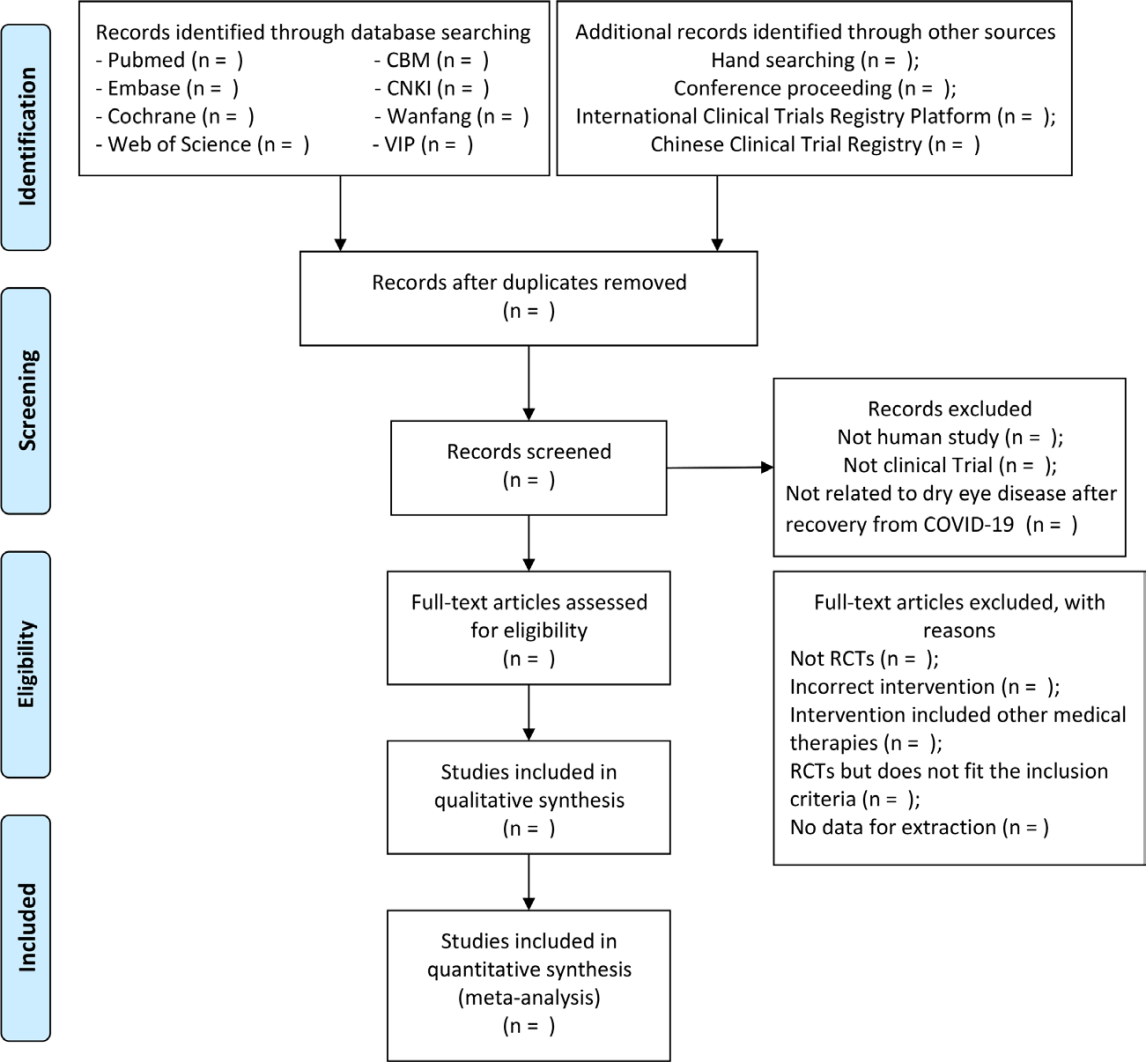
PRISMA flow chart of study selection process. PRISMA = preferred reporting items for systematic reviews and meta-analyses.

Two independent reviewers will screen and evaluate the relevant abstracts and titles of all studies against pre-determined inclusion criteria, then exclude duplicates or unqualified articles and explain why. A third investigator will resolve any differences between the 2 examiners. The process for filtering selections is shown in Figure 1.

#### 2.4.2. Data extraction and management.

Two reviewers will be responsible for the extraction and management of data according to the retrieval strategy, including study title, journal, year of publication, name of first author, general information, study design, experimental intervention and timing of intervention, results, and adverse events. If there is any disagreement between the 2 reviewers during the data extraction process, the panel will jointly arbitrate and make a decision.

#### 2.4.3. Dealing with missing data.

If complete literature or relevant data is not available, we will contact the corresponding author. However, if the missing data cannot be obtained, then the study will be excluded from the analysis.

#### 2.4.4. Assessment of risk of bias.

The Cochrane Handbook for Systematic Reviews of Interventions Version 6 will be performed to assess a broad category of biases in the included studies. We will evaluate biases from the following 7 aspects: random sequence generation, allocation concealment, blinding of the participants and personnel, blinding of the outcome assessments, incomplete outcome data, selective reporting, and other sources of bias. These studies will be assigned as low risk, high risk, or unclear risk. Inconsistencies will be resolved by discussion with other reviewers.

#### 2.4.5. Measures of treatment effect.

Review Manager (RevMan 5.3, Cochrane Collaboration, Nordic Cochrane Center, Copenhagen, Denmark) software and Stata 14.2 (Stata Corp, College Station, TX) will be used to conduct this metaanalysis. Dichotomous outcomes will be presented as risk ratios with 95% confidence intervals. When continuous outcomes exist, mean differences or standardized mean differences will be calculated.

#### 2.4.6. Assessment of heterogeneity.

Cochrane *χ*^2^ and *I*^2^ tests will be used for the evaluation of heterogeneity. It is acknowledged that if *P* ≥ .05 and *I*^2^ ≤ 50%, the assessment of heterogeneity can be neglected; and there is great heterogeneity between included studies if *P* < .05 and *I*^2^ > 50%.

#### 2.4.7. Assessment of reporting bias.

If there are over 10 studies included in the meta-analysis, funnel plots will be used to detect the reporting biases.^[11]^

#### 2.4.8. Data synthesis.

We will take advantage of Review Manager (RevMan) software V.5.3 for data analysis and synthesis. Data will be processed with a fixed-effect model if no statistical heterogeneity was observed among the results (*P* ≥ .05 and *I*^2^ ≤ 50%). Meanwhile, the random-effect model will be put into use, if *P* < .05 and *I*^2^ > 50%.

#### 2.4.9. Subgroup analysis.

Based on the results of data synthesis, a subgroup analysis or meta-regression analysis will be performed to analyze the source of any heterogeneity.

#### 2.4.10. Sensitivity analysis.

Sensitivity analysis will be performed to examine the robustness of the study’s conclusions. Will include methodological quality, sample size, and the impact of missing data. Therefore, the impact of low-quality studies on overall results will be assessed.

#### 2.4.11. Quality of evidence evaluation.

The quality of evidence will be independently assessed by 2 reviewers and graded for recommendation evaluation, development and evaluation. Evidence quality will be rated as “high,” “medium,” “low,” or “very low” according to rating criteria based on 5 parameters (publication bias, inconsistencies, inaccuracies, and research limitations). Differences during this process will be decided through negotiation or by the third senior researcher.

#### 2.4.12. Ethics and dissemination.

Given that this protocol is for a systematic review which involves no privacy data, ethical approval and informed consent are needless. The results of this review will be disseminated widely through being submitted to peer-reviewed publications and conference presentations.

## 3. Discussion

DED is the most common clinical ocular surface disease at present. The causes of DED are complicated. The survey shows that the global prevalence of DED is 5% to 50%,^[3]^ and the incidence of DED in China is as high as 21.0% to 30.0%,^[12]^ which can cause significant vision loss and thus affect its normal work and life.

Acupuncture for the treatment of dry eyes is in the Angle of traditional Chinese medicine Play the role of mediating Yin and Yang, acupuncture as a long history traditional Chinese medicine treatment has the advantages of few adverse events and no drug dependence, Combining modern medical technology and theory has achieved good curative effect.^[4]^ It is necessary to conduct a systematic review to establish convincing evidence for evaluating the effectiveness and safety of acupuncture for DED after recovery from COVID-19. Therefore, we will adopt a more rigorous systematic evaluation method to provide evidence-based data for the treatment of DED after recovery from COVID-19 with the method of acupuncture and provide new ideas and methods for the treatment and research of DED after recovery from COVID-19.

## Author contributions

**Data curation:** Xiaofang Lu, Jian Li, Shuhan Ye.

**Funding acquisition:** Yijun Chen.

**Writing – original draft:** Runyi Wang, Yao Xiao, Lu Liu, Wanning Lan, Zongli Liao.

**Writing – review & editing:** Xiaofang Lu, Jian Li, Shuhan Ye, Zongli Liao, Yijun Chen.
